# Reverse-Phase Ultra-Performance Chromatography Method for Oncolytic Coxsackievirus Viral Protein Separation and Empty to Full Capsid Quantification

**DOI:** 10.1089/hum.2022.013

**Published:** 2022-07-13

**Authors:** James Z. Deng, Richard R. Rustandi, Damon Barbacci, Andrew R. Swartz, Amanda Gulasarian, John W. Loughney

**Affiliations:** ^1^ Vaccine Analytical Research & Development, Merck & Co., Inc., Kenilworth, New Jersey, USA.; ^2^ Vaccine Process Research & Development, Merck & Co., Inc., Kenilworth, New Jersey, USA.

**Keywords:** oncolytic Coxsackievirus, RP-UPLC, capsid empty/full ratio, capsid quantification, therapeutic viral vector, LC/MS, virion protein analysis, V937

## Abstract

Oncolytic virus immunotherapy is emerging as a novel therapeutic approach for cancer treatment. Immunotherapy clinical drug candidate V937 is currently in phase I/II clinical trials and consists of a proprietary formulation of Coxsackievirus A21 (CVA21), which specifically infects and lyses cells with overexpressed ICAM-1 receptors in a range of tumors. Mature Coxsackievirus virions, consisting of four structural virion proteins, (VPs) VP1, VP2, VP3, and VP4, and the RNA genome, are the only viral particles capable of being infectious. In addition to mature virions, empty procapsids with VPs, VP0, VP1, and VP3, and other virus particles are produced in V937 production cell culture. Viral protein VP0 is cleaved into VP2 and VP4 after RNA genome encapsidation to form mature virions. Clearance of viral particles containing VP0, and quantification of viral protein distribution are important in V937 downstream processing. Existing analytical methods for the characterization of viral proteins and particles may lack sensitivity or are low throughput. We developed a sensitive and robust reverse-phase ultra-performance chromatography method to separate, identify, and quantify all five CVA21 VPs. Quantification of virus capsid concentration and empty/full capsid ratio was achieved with good linearity, accuracy, and precision. ClinicalTrials.gov ID: NCT04521621 and NCT04152863.

## INTRODUCTION

Oncolytic viruses that target specific cancer cell types have been developed as novel immunotherapy approaches. Oncolytic viruses that specifically target and destroy tumor cells and enhance the adaptive immune response are considered as therapeutic tools with great potential.^1,2^ V937 is an oncolytic virotherapy clinical drug candidate consisting of an oncolytic strain of Coxsackievirus A21 (CVA21). It has been studied in a range of tumors that upregulate cell surface receptor ICAM-1, including melanoma, bladder cancer, and breast cancer. In early clinical trials, V937 demonstrated viral targeted tumor cell death with promising clinical outcomes and acceptable safety profiles.^3–5^

CVA21 belongs to the genus enterovirus within the family Picornaviridae. Other viruses in the family Picornaviridae include Poliovirus and Rhinoviruses. Five capsid structural virion proteins (VPs) are generated from precursor polyprotein in Coxsackievirus morphogenesis. These VPs are originated from the P1 domain of the precursor polyprotein. A myristoylated (Myr) VP0-VP1-VP3 heterotrimeric promoter complex is initially formed. These Myr-VP0-VP1-VP3 protein complexes can be assembled into an empty procapsid through pentamer intermediates.

**Figure 1.**
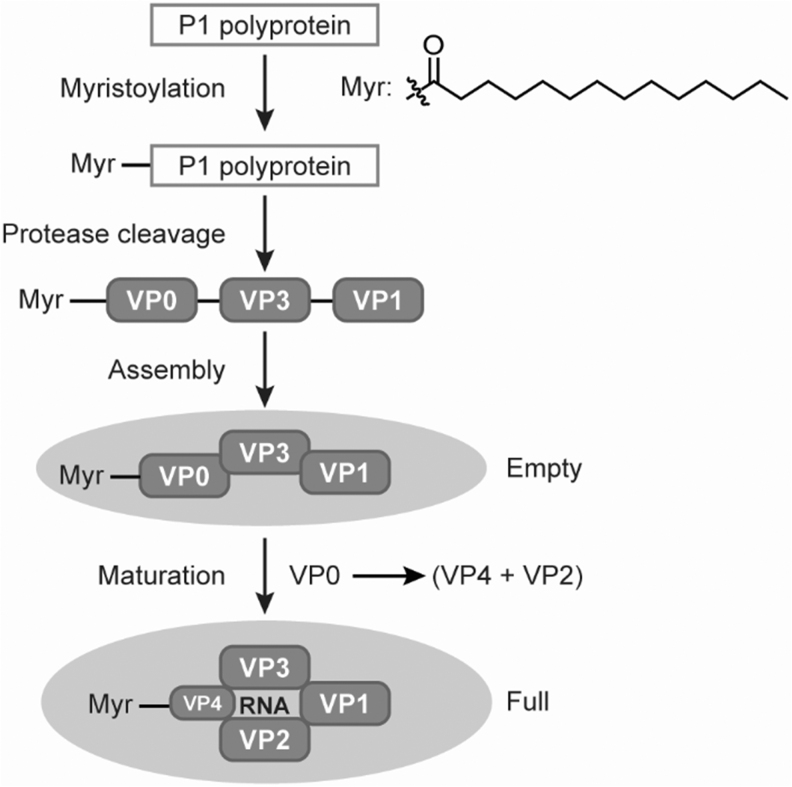
Coxsackievirus VPs and virus capsids formation. VPs, virion proteins.

Each empty capsid contains 12 pentamers or 60 copies of individual VP0, VP1, and VP3. When the virus genome RNA is encapsidated by the pentameric protein complexes, an intermediate provirion is formed first, but then converted to a full mature virion upon cleavage of VP0–VP2 and VP4. Each full capsid will contain 60 copies of individual VP1, VP2, VP3, and VP4, plus a VPg-modified single-stranded RNA (7.4 kb) genome (Fig. 1).^6–10^ Both empty and full capsids of Coxsackievirus appear in similar sizes with diameters of ∼30 nm.^9,11^

Generally, mature, full virions are the only particles capable of eliciting oncolytic activity. The empty capsids may generate undesired immune response that could compromise oncolytic potency of the product. Characterization of virus VPs and quantitation of virus capsid titers or concentrations (capsids/mL or Cp/mL) are of great interest to the gene therapy and vaccine research and product development.^12–14^ Accurate and precise measurements of capsid concentrations and capsid empty/full ratio are required to define clinical dosage and product target profile. Conventionally, the virus capsid concentration and/or capsid empty/full ratio are measured using low-throughput techniques, such as analytical ultracentrifugation, Cryo-EM.^15–17^ Polymerase chain reaction techniques that measure the genome titer or immuno-binding assays, such as ELISA, have also been employed to assess oncolytic particles. These assays are highly variable and labor intensive.^18,19^

 Batch mode optical density (OD) measurements have been used to measure the product in bulk. In the OD measurements, the target virus is not separated from unwanted impurities and sample matrix. The batch OD measurements can easily encounter accuracy or/and precision issues, due to the heterogeneity nature of the virus products and lack of assay specificity.^20,21^ Recently, analytical ion exchange chromatography/high performance liquid chromatography (HPLC) method for adeno-associated virus (AAV) has also been developed with success highly dependent on the consistency of the virus surface charges.^22,23^ For AAVs, size-exclusion chromatography (SEC) methods involving multiple in-line detectors were also reported recently,^24–26^ as well as capillary isoelectric focusing and native mass spectrometry.^27–29^ We have recently reported SEC methods coupled with in-line multiple detectors for the characterization of our V937 oncolytic Coxsackievirus and other vaccines,^11,30^ but there is no report that uses reverse-phase chromatography to quantify virus empty/full capsid ratio.

Because VP2 and VP4 are cleaved from VP0 without losing any amino acid residue,^31^ the total protein content is considered to remain constant for empty procapsid, provirion, and full mature virion. Therefore, the total capsid concentration of a virus sample can be deduced from measured total protein concentration, when compared with a virus standard of known capsid concentration. Furthermore, the moles of VP0 in an empty capsid is equal to the moles of VP2 or VP4 in a full capsid. Mature full capsids contain VP2 and VP4 without VP0, whereas empty provirion capsids contain VP0, but not VP2 and VP4. Based on the capsid's structural features, the VP0/(VP2+VP4) peak area ratio can be used to represent capsid empty/full ratio. Herein, we have developed a robust and sensitive reverse-phase ultra-performance chromatography (RP-UPLC) assay that can separate, identify, and quantify all five Coxsackievirus VPs (VP0, VP1, VP2, VP3, VP4) in a single run.

Both VP0 and VP4 were observed as myristoylated proteins. Since the sum of total VPs is consistent for empty and full capsids, a pure empty or full capsid reference standard was not required for capsid (Cp) quantification. Virus samples with known capsid concentration are suitable for assay reference standards, even if they might be a mixture of empty and full capsids. This can be advantageous for early process development, when a pure empty or full capsid standard is not easily available. This assay has demonstrated good linearity, accuracy, and precision for quantification of capsid particle concentration and capsid empty/full ratio.

## EXPERIMENTAL

### Materials

Around 0.1% trifluoroacetic acid (TFA) HPLC water and 0.1% TFA acetonitrile were purchased from MilliporeSigma (St. Louis, MO). Bovine serum albumin (BSA) standard ampules (2 mg/mL) and dithiothreitol (DTT) were purchased from Thermo Fisher (Waltham, MA). BIOshell IgG C4 columns (1,000 Å, 2.1 × 100 mm, 2.7 μm) were purchased from MilliporeSigma.

### V937 drug substance production

Briefly, V937 drug substance (DS) was produced from infected MRC-5 (derived from ECACC 05072101) cell culture using a virus seed derived from a CVA21 Kuykendall prototype strain (ATCC VR-850). Lysates were harvested and clarified through depth filters and purified across an affinity chromatography and two polishing ion exchange chromatography steps to concentrate and clear residual impurities and procapsids. Purified V937 was exchanged into a stabilizing buffer and passed through a 0.2 μm filter to generate the final DS. Empty procapsids and empty/full capsid mixtures were purified from an intermediate chromatography step. Three batches of Process 1 and Process 2 sample intermediates were generated using different process conditions and analyzed by the RP-UPLC method.

### RP-UPLC separation

The RP-UPLC was performed on a Waters ACQUITY UPLC system (Waters Corporation, Milford, MA), including a quaternary (or binary) pump, sample manager, column component, fluorescence (FLR), and tunable UV or photodiode array detectors. The FLR was detected using excitation wavelength at 280 nm and emission wavelength at 352 nm. UV absorbance at 280 nm (A280) and 220 nm (A220) were also monitored. A BIOshell IgG C4 column (1,000 Å, 2.1 × 100 mm, 2.7 μm) from MilliporeSigma was used at a column temperature maintained at 80°C.

The linear gradient elution (Table 1) was formed by Mobile phase A, consisting of 0.1% TFA in HPLC or liquid chromatography–mass spectrometry (LC/MS) grade water, and Mobile phase B, consisting of 0.1% TFA in acetonitrile with the flow rate of 0.4 mL/min. The sample manager temperature was maintained at 8^°^C during analysis. V937 samples were injected directly without modifications, unless noted in the context. Chromatograms were processed with Waters Empower 3 software for peak integration. Signals from the FLR channel (*Ex* 280 nm, *Em* 352 nm) were used for all quantification.

**Table 1.** Reverse-phase ultra-performance chromatography elution gradient

Time (min)	Flow Rate (mL/min)	%A	%B
0	0.4	75	25
0.5	0.4	75	25
4.5	0.4	58	42
10.5	0.4	52	48
11.5	0.4	20	80

### LC/MS analysis of intact VPs

LC/MS experiments were performed on a Waters ACQUITY UPLC coupled with a Waters Xevo G2-XS Q-TOF (Quadrupole Time-of-Flight) mass spectrometer (Waters Corporation). The MilliporeSigma BIOshell IgG C4 column (1,000 Å, 2.1 × 100 mm, 2.7 μm) was also used for LC/MS peak separation. LC/MS was run in a similar fashion as RP-UPLC (Table 1) with modified mobile phases to be more compatible with mass spectrometry detector. In LC/MS, mobile phase A consists of 0.1% formic acid and 0.025% TFA in LC/MS grade water, whereas mobile phase B consists of 0.1% formic acid and 0.025% TFA in acetonitrile.

Mass spectra were obtained in positive mode by spraying the eluent into the mass spectrometer using an electrospray ionization source. The capillary, source cone, and extraction cone voltages were set at 3 kV, 50 V, and 80 V, respectively. Nitrogen was used as a desolvation gas at a flow rate of 600 L/h. The source and desolvation temperatures were set at 120°C and 400°C, respectively. The instrument was operated in Sensitivity mode and spectra were acquired in an *m/z* range of 400–3,000. Data acquisition and analysis (deconvolution) were performed with Waters (MassLynx 4.1 software). Protein spectra were deconvoluted to obtain the observed intact protein masses. A uniform Gaussian model was used with width at half height of either 1 or 0.8.

## RESULTS AND DISCUSSION

### RP-UPLC separation and identification of VPs0, VPs1, VPs2, VPs3, VPs4, on LC/MS

The V937 DS virus capsids have a diameter of ∼30 nm.^11^ At the beginning of the method development, we sought to identify a column of large-pore beads that can accommodate the capsids and allow on-column capsid dissociation to individual VPs from the capsid and separation within the pores of stationary beads. After extensive column screenings, the MilliporeSigma BIOshell IgG C4 column (1,000 Å, 2.1 × 100 mm, 2.7 μm) was selected from a collection of columns, such as Agilent AdvancedBio RP-mAb C4, Waters Protein BEH C4, YMC-Pack PROTEIN-RP, and Tosoh TSKGel Protein C4-300. This column offers better peak shape, resolution, and recovery than other columns that were screened, presumably due to the large pore size (1,000 Å) of the column stationary phase that allows the virus capsid enter into the beads, and get disassembled inside the pore into each VPs for separation.

After initial success of separating VP1, VP2, VP3, and VP4 for V937 DS on an Agilent 1260 HPLC system, the method was optimized on a Waters UPLC system to improve separation efficiency (Supplementary Figs. S1 and S2, supplemental information). The UPLC method showed much improved peak resolutions (Supplementary Table S1a and b, supplemental information). DTT-treated V937 DS showed unchanged peak pattern when compared with the untreated sample injected directly on the RP-UPLC (Supplementary Fig. S3, supplemental information). This suggests that there are no disulfide bonds between VPs.

The peaks on the chromatogram were initially assigned, based on the calculated physicochemical properties listed on Supplementary Table S2 (supplemental information) from known V937 capsid structure and VP sequence information.^11,29,32^ These were confirmed later by mass detected for each peak on LC/MS and supported the earlier hypothesis that the capsids are disassembled on column to each individual VPs. The highly negative charged and polar genome RNA is expected to elute out quickly close to the void volume of the RP-UPLC column.

**Figure 2.**
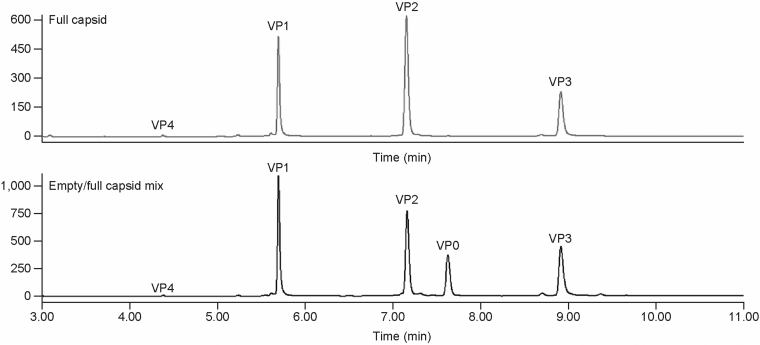
Comparison RP-UPLC chromatograms of VP profile from purified full capsids with that from mixture empty/full capsids. VP0 is not visible in the purified full capsids. RP-UPLC, reverse-phase ultra-performance chromatography.

To separate and identify all five Coxsackievirus VPs (VP0, VP1, VP2, VP3, and VP4) on the chromatogram, an intermediate process sample containing a mixture of empty and full capsids was generated. A distinct extra peak was detected between VP2 and VP3 in comparison to peaks from purified full capsid (Fig. 2 and Supplementary Fig. S8 in supplemental information). After the samples were analyzed on LC/MS (Table 2 and Supplementary Fig. S4 in supplemental information), the peaks were assigned by mass to the corresponding VP0, VP1, VP2, VP3, and VP4 as in Fig. 2.

**Table 2.** Identification of Coxsackievirus virion proteins by liquid chromatography–mass spectrometry

VPs	VP0	VP1	VP2	VP3	VP4
Calculated Mw from amino acid sequence (Da)	37,183	33,231	29,897	26,546	7,304
Mass observed on LC/MS (Da)	37,418	33,230	29,922	26,591	7,516
Observed—calculated Mw (Da)	235	−1	25	45	212
Peak assignment	(VP0+Myr) +Na^+^	VP1	VP2+Na^+^	VP3 + 2Na^+^	VP4+Myr

LC/MS, liquid chromatography–mass spectrometry; Mw, molecular weight; VPs, virion proteins.

**Figure 3.**
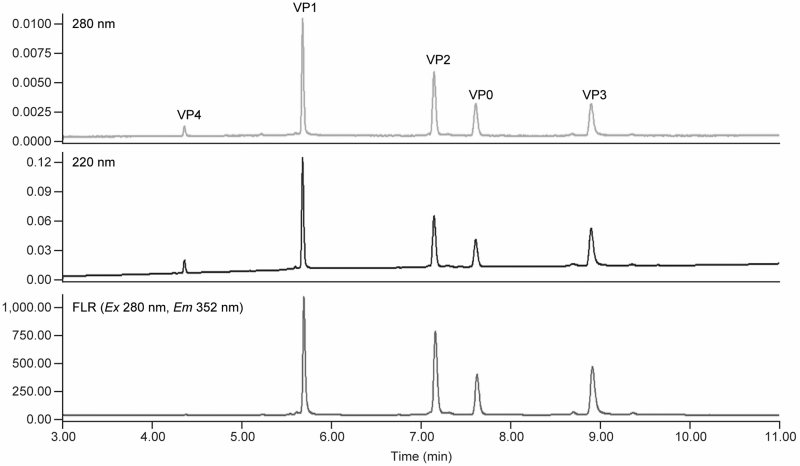
Overlay of A220, A280 and FLR signals for all five Coxsackievirus VPs. FLR, fluorescence.

The RP-UPLC separation method was developed through an iterative optimization process. Comparison of the virion peak intensity/sensitivity on the three detection channels (UV A220, A280, and FLR) (Fig. 3) led to the decision to use FLR channel for peak quantitation due to its much higher detection sensitivity. VP4 not only has much shorter amino acid sequence and lower molecular weight (Mw) than the rest of VPs, but also has much lower calculated aliphatic index and hydropathicity (Supplementary Table S2, supplemental information). It is expected to elute out much earlier than the rest of VPs on RP-UPLC. Based on these and earlier experience on HPLC method development, a two-stage elution gradient was employed (Table 1). The first stage was 25–42%B for 4 min with VP4 eluting at the end of the first-stage gradient. The second stage, a slower gradient from 42% to 48%B for 6 min, was used for separating the remaining four VPs.

**Figure 4.**
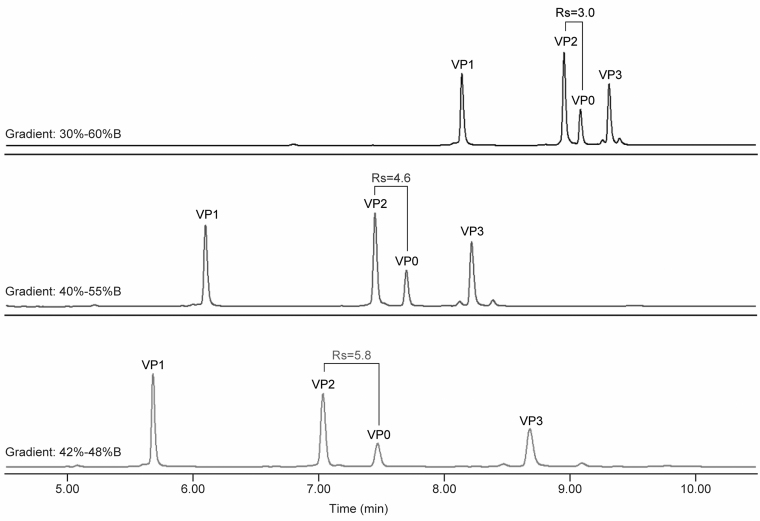
Optimization of elution gradient to improve Rs. Rs, peak resolution.

The second stage was the focus of UPLC method optimization. The second-stage gradient was arrived by gradually attenuating the gradient slope to achieve better peak resolution (Rs). The peak resolution between VP2 and VP0 was the main focus for method development, because of similar peak retention times due to their highly overlapped amino acid sequence and the FLR VP0/VP2 ratio was later selected to monitor capsid empty/full ratio in process development. The gradient optimization is shown in Fig. 4 and in Table 3. The VP2/VP0 Rs value of 5.8 well exceeded the Rs value between the peak of interest required by regulatory agency for method validation (Rs >2).^33^ Peak resolutions between other VPs pairs were at least two- to four-fold better than the VP2/VP0 peak resolution.

**Table 3.** Gradient optimization to improve virion protein2/VP0 peak resolution

Second stage gradient	Rs		
	VP1/VP2	VP2/VP0	VP0/VP3
30–60%B	18.6	**3.0**	5.2
40–55%B	25.6	**4.6**	9.4
42–48%B	23.1	**5.8**	14.0

Numbers in bold show the peak resolution between VP2 and VP0.

Rs, peak resolution.

Based on knowledge of the capsid starting to disassemble at ∼60°C,^11^ column temperatures from 70°C to 85°C were selected for optimization. In all temperatures tested, good peak resolutions were observed (Supplementary Fig. S5 and Supplementary Table S3, supplemental information). Flow rates from 0.35 to 0.425 mL/min were also explored. The two higher flow rates, 0.425 mL/min and 0.40 mL/min, showed a little better peak resolution (Supplementary Fig. S6 and Supplementary Table S4, supplemental information). In both cases, the assay showed good robustness against fluctuations in column temperature and flow rate. The column temperature of 80°C and flow rate of 0.40 mL/min were selected for balanced chromatogram profiles and room for assay robustness.

With these, the UPLC method in combination with LC/MS offer separation and identification of all five CVA21 VPs. The viral capsids were disassembled into each individual VPs under the chromatography elution condition, and there is no covalent crosslink between VPs or subunits. The on-column capsid disassembly eliminated the need for tedious sample preparations before analysis. These sample preparations could alter properties of the VPs and result in recovery issues.

Mws for four of the five VPs are ranged within a window of only ∼10 kDa wide (27–37 kDa). For the proteins with size ranging in such a narrow window, achieving good resolution using a size-based separation method can be challenging. Exploiting differences in protein hydrophobicity, the RP-UPLC method demonstrated good separation efficiency. Process impurities such as serum or host cell proteins would have different amino acid sequences from VPs. This would result in some hydrophobicity differences that can be explored for RP-UPLC separation. This was demonstrated by BSA protein separation from all VPs on the chromatogram in a capsid sample spiked with BSA (Supplementary Fig. S7, supplemental information).

Both VP4 and VP0 appeared as myristic acid adducted proteins on LC/MS, with Mw of a naked protein plus a myristoyl (Myr) side chain (Table 2). Previously, myristoylation was only observed in X-ray structural study of intact enteroviruses or by incorporating of radiolabeled Myr into viruses.^6,34,35^ The myristoyl side chain plays an important role in virus maturation and host cell interactions.^36–38^ Inhibition of myristoylation by siRNA knockdown and use of myristic acid analogs prevented cleavage between VP4 and VP2 as well as reduction in viral RNA synthesis.^39,40^ Direct separation and identification of Myr-adducted VP0 and VP4 offer opportunities for more structural and physiochemical characterization for these species. Combined information from chromatography and mass spectroscopy would help future product and process understanding and investigations.

### Capsid concentration and empty/full ratio quantification

Coxsackievirus maturation involves a critical protease cleavage step that converts VP0–VP2 and VP4. All matured full capsids have (VP2+VP4), but not VP0. VP2 and VP4 are absent from empty capsids, where VP0 is presented. The amino acid residues remain the same when VP0 is cleaved into (VP2+VP4). Since the VP UV and FLR peaks represent the amino acid sequence of the protein under the denatured conditions on the RP-UPLC, we can reasonably propose to use VP0/(VP2+VP4) peak ratio to measure capsid empty/full ratio. In the FLR detection channel, since VP4 is a much smaller protein without a tryptophan residue (Supplementary Table S2, supplemental information), its contribution to the (VP2+VP4) FLR peak area is negligible (VP4/VP2 < 1% in FLR peak area). So VP0/VP2 FLR peak ratio can be used directly as a simplified term to quick assess empty/full ratio in process development.

From the same structure information, the total amino acids remain the same between a full capsid and an empty capsid. Therefore, the sum of VP peak areas from a sample can be used to quantify sample total capsid concentration (capsids/mL) when compared with a reference standard of known capsid concentration.

#### Linearity and range

**Figure 5.**
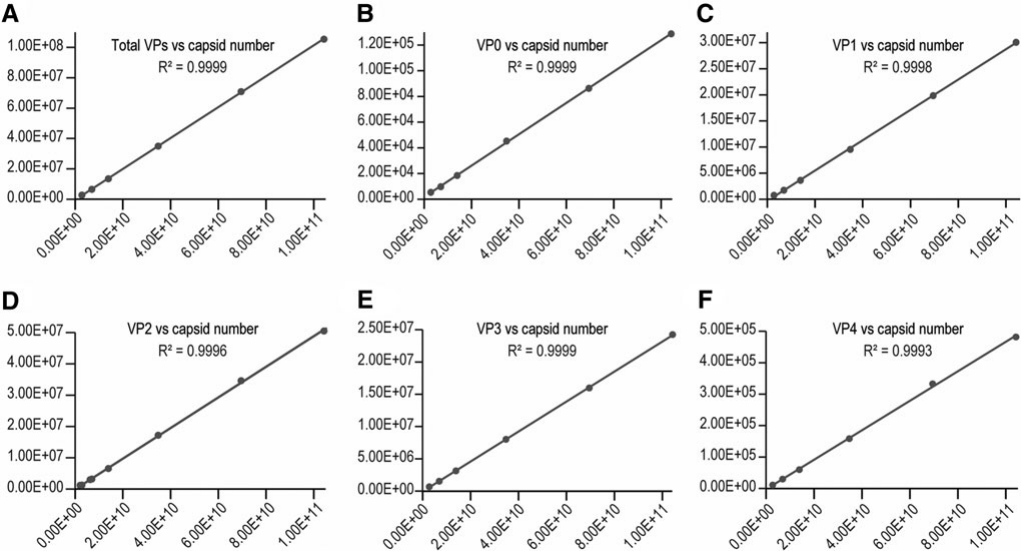
**(A–F)** Linearity curves from a virus standard.

Linearity was first established using a V937 virus standard sample with known capsid concentration of 1.39E12 capsids/mL. A linear standard curve was generated using peak areas from the UPLC FLR channel versus the injected particle number. Good linearity (*R*^2^ > 0.999) has been observed for each VP and sum of all VPs (Fig. 5 and Supplementary Table S5 in supplemental information). The particle number in the standard curve ranged from 2.78E9 to 1.04E11 capsids per injection. The intercept and slope from the standard curve of total VP peak area are used to calculate the capsid numbers from a virus sample injection in Eq. (1).

(1) $$\begin{array}{cc} & Capsidnumberperinjection\\ & =\frac{\left(TotalVPpeakarea-Intercept\right)}{Slope} \end{array}$$

The particle concentration (/mL) of a virus sample can be calculated from Eq. (2):

(2) $$\begin{array}{cc} & Capsidconcentration\left(Capsids∕mL\right)\\ & =\frac{Capsidnumberperinjection}{\left(Injectionvolume\left(mL\right)∕Dilutionfactor\right)} \end{array}$$

**Figure 6.**
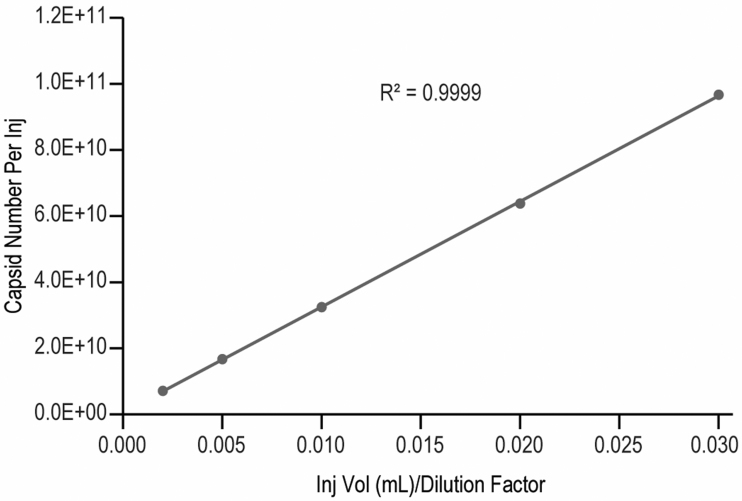
Virus sample dilutional linearity curve.

Besides the linearity for the standard, a good sample dilutional linearity is often required by regulatory agency to demonstrate the accuracy of measurement for a bioanalytical method, especially for samples that a good spike standard is hard to be defined or obtained.^41^ In this case, we analyzed our virus sample-A at five dilution levels (neat, 1.5 × , 3 × , 6 × , 15 × ) within the standard curve range. The capsid number per injection was calculated from total VP peak area by using the slope and intercept obtained from the standard curve (Supplementary Table S5 in supplemental information) and Equation-1. Not only did the measured particle number per injection showed good linearity (*R*^2^ = 0.9999) against the dilution factor/corrected injection volume (injection volume/dilution factor), but also good linearity has been observed for each individual VP (Fig. 6 and Supplementary Table S6 in Supporting information).

The sample capsid concentration (capsids/mL) calculated from Eq. (2) also showed good consistency across the dilutional levels with relative standard deviation (%RSD) <5% (Supplementary Table S6, supplemental information). These demonstrate that the RP-UPLC assay has good linearity and is consistent across different concentrations.

#### Precision and repeatability

A research sample (Sample-1) was analyzed on two systems for precision and repeatability, with triplicate injections on UPLC each time. The retention time of each VP in these samples are constant with %RSD <1% (Table 4). The sample also showed good precisions on peak areas from each VPs and total proteins, as well as the VP0/(VP2+VP4) ratio to represent the capsid empty/full ratio in the samples. As a result, the total capsid particle concentration for the sample was measured with good precision (%RSD = 0.8, Table 5). Two other V937 samples (Sample-2 and Sample-3) were also measured in triplicate injections, in which both demonstrated good precisions (Supplementary Tables S7 and S8, supplemental information).

**Table 4.** Precision of peak retention time

Sample-1	INJ	RT (min)				
		VP4	VP1	VP2	VP0	VP3
Exp-1/Sys-1	INJ-1	4.336	5.669	7.076	7.523	8.769
	INJ-2	4.334	5.669	7.088	7.536	8.782
	INJ-3	4.336	5.67	7.073	7.522	8.775
	Avg	4.335	5.669	7.079	7.527	8.775
	%RSD	0.03	0.01	0.11	0.1	0.07
Exp-2/Sys-2	INJ-1	4.374	5.696	7.163	7.627	8.911
	INJ-2	4.37	5.691	7.158	7.624	8.913
	INJ-3	4.375	5.697	7.171	7.641	8.924
	Avg	4.373	5.695	7.164	7.631	8.916
	%RSD	0.06	0.06	0.09	0.12	0.08
Avg		4.354	5.682	7.122	7.579	8.846
%RSD		0.48	0.25	0.66	0.76	0.87

%RSD, relative standard deviation; Exp, experiment; INJ, injection; RT, retention time; Sys, system.

**Table 5.** Precision of peak area

Sample-1		FLR peak area							Empty/Full	(Capsid) (/mL)
		VP4	VP1	VP2	VP0	VP3	Total of all VPs	VP2+VP4	VP0/(VP2+VP4)	
Exp-1/Sys-1	INJ-1	193,564	22,588,588	21,890,324	11,698,177	17,846,286	74,216,939	22,083,888	0.53	3.66E+12
	INJ-2	193,956	22,455,981	22,072,816	11,671,564	17,881,673	74,275,990	22,266,772	0.524	3.67E+12
	INJ-3	200,811	22,184,617	22,064,813	11,447,195	17,769,682	73,667,118	22,265,624	0.514	3.64E+12
	Avg	196,110	22,409,729	22,009,318	11,605,645	17,832,547	74,053,349	22,205,428	0.523	3.66E+12
	%RSD	2.08	0.92	0.47	1.19	0.32	0.45	0.47	1.51	0.45
Exp-2/Sys-2	INJ-1	179,236	22,026,203	21,541,602	11,979,694	17,320,598	73,047,333	21,720,838	0.552	3.61E+12
	INJ-2	183,989	22,140,644	21,649,730	12,036,562	17,416,280	73,427,205	21,833,719	0.551	3.63E+12
	INJ-3	186,503	21,690,748	21,647,757	12,123,676	17,060,923	72,709,607	21,834,260	0.555	3.59E+12
	Avg	183,243	21,952,532	21,613,030	12,046,644	17,265,934	73,061,382	21,796,272	0.553	3.61E+12
	%RSD	2.01	1.07	0.29	0.6	1.06	0.49	0.3	0.4	0.49
Average		188,757	22,148,473	21,782,867	11,857,645	17,508,768	73,486,511	21,971,625	0.54	3.63E+12
%RSD		4.2	1.4	1.1	2.2	1.9	0.9	1.1	3.2	0.8

FLR, fluorescence.

#### Assay accuracy

All of our V937 DSs are highly purified full capsids with <1% empty particles. Our standard has ∼0.25% empty capsids with a capsid concentration of 1.39E12 (capsids/mL). To assess assay accuracy on both capsid concentration and empty/full ratio, a less purified research batch (Sample-B) with higher empty/full ratio was generated. This material has capsid concentration of 3.66E12 (capsids/mL) and 34.3% empty capsid within the sample (Supplementary Table S9, supplemental information). The standard was mixed with sample-B in four different ratios (Supplementary Table S10, supplemental information) to probe the changes in both particle concentration and empty/full ratio. We focused on empty/full ratio change in samples with low empty capsid contents (Mix-2, Mix-3, and Mix-4), since these represent most likely cases from V937 DS samples. Theoretical values for each mix sample were calculated out based on the inputs from the standard and sample-B (Supplementary Table S11, supplemental information).

Each mixed sample was injected in triplicate on a RP-UPLC. The averaged peak areas of the three injections were used to calculate measured particle concentration and empty/full ratio from a standard curve (Supplementary Table S12, supplemental information). The measured capsid concentration and empty/full ratio were compared with corresponding theoretical values and summarized in Table 6. The measured total particle concentration was within 102–106% of the theoretical, and the measured empty/full ratio was within 90–100%. These indicate that the assay has good accuracy for both total particle concentration and empty/full ratio. The expected change of empty/full ratio from 0.036 of Mix-3 to 0.024 of Mix-4 was measured with good accuracy. This demonstrates that the assay has the sensitivity to measure change of 0.012 (1.2%) in empty/full ratio.

**Table 6.** Assay accuracy

Spiked sample	Theoretical values		Measured values		% (Measured/theoretical)	
	(Total capsid) (/mL)	Empty/full ratio	(Total capsid) (/mL)	Capsid empty/full ratio	Total capsid	Empty/full ratio
Mix-1	2.52E+12	0.3323	2.58E+12	0.3276	102	99
Mix-2	1.60E+12	0.0793	1.68E+12	0.0715	105	90
Mix-3	1.48E+12	0.0362	1.57E+12	0.0362	106	100
Mix-4	1.45E+12	0.0241	1.52E+12	0.0237	105	98

### Process monitoring and optimization

**Figure 7.**
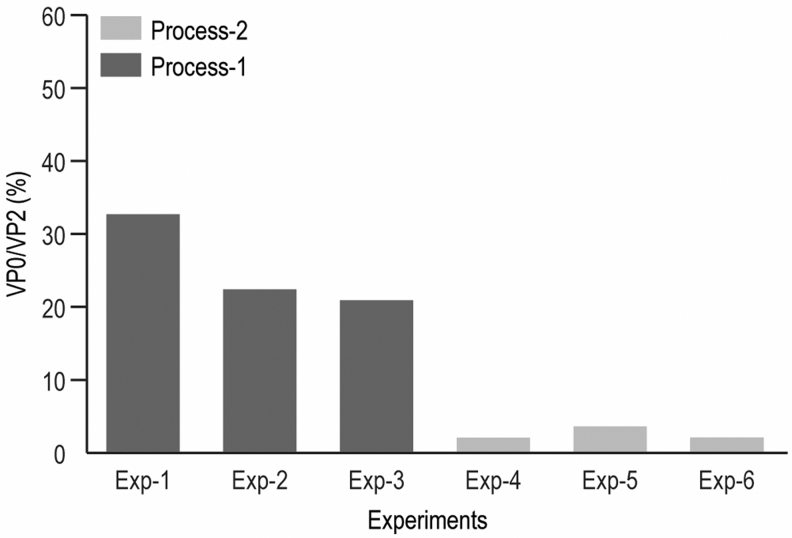
Comparison of VP0/VP2 ratios from two different processes.

One critical application of this RP-UPLC assay is the quantification of empty/full capsid ratio for downstream process monitoring and optimization. In the process monitoring, VP0/VP2 FLR peak ratio is used to measure capsid empty/full ratio in the sample. Purification intermediate samples from three experiments in each of Process-1 and Process-2 were monitored. VP0/VP2 ratios were more than 20% for three experiments using Process-1, indicating undesired high empty content in the intermediates and a less optimal process. After further process optimization, VP0/VP2 was decreased to <4% for all three Process-2 experiments, indicating improved empty capsid clearance (Fig. 7 and Supplementary Table S13 in supplemental information). The VP0/VP2 ratio was evaluated for multiple V937 DS batches and the percentage of empty particle in V937 DS samples was <1% in all batches (Supplementary Table S14, supplemental information). These data indicate that the V937 DS consists of purified full mature virions with VP0 cleaved and highlights the utility of the RP-UPLC in both DS characterization and process development.

## CONCLUSION

Guided by the structural information of the virus and VPs, we developed a rapid and efficient RP-UPLC assay for characterization and quantitation of oncolytic Coxsackievirus capsids and VPs. Coupled with mass spectrometry, this method not only can identify each individual VPs, but also reveal the characteristic structural features of the VPs, such as the myristoyl adduction on VP0 and VP4. The fact that Coxsackievirus VPs are separated and identified for the first time on the chromatography can open doors for more biochemical and biophysical studies of the virus and its VPs. Reverse-phase chromatography is used for the first time to quantify a viral capsid empty/full ratio, a critical attribute that impacts the quality and clinical dosage of viral vectors and gene therapy products. Success of this structure-guided chromatography method development approach will stimulate the interest of using unique structural features of virus and viral vectors for future assay development.

The use of FLR detection offers great sensitivity enhancement versus most current methods that use UV or/and refractive index for detection. In this assay, both virus capsid concentration and capsid empty/full ratio are quantified with good sensitivity, linearity, precision, and accuracy. This quantification strategy can be expanded to other viruses, such as poliovirus, human rhinovirus, and foot-and-mouth disease virus or engineered viral vectors with similar capsid structures.

## Supplementary Material

Supplemental data

Supplemental data

Supplemental data

Supplemental data

Supplemental data

Supplemental data

Supplemental data

Supplemental data

Supplemental data

Supplemental data

Supplemental data

Supplemental data

Supplemental data

Supplemental data

Supplemental data

Supplemental data

Supplemental data

Supplemental data

Supplemental data

Supplemental data

Supplemental data

Supplemental data
