# Supplementary figures and images for: Reverse-Phase Ultra-Performance Chromatography Method for Oncolytic Coxsackievirus Viral Protein Separation and Empty to Full Capsid Quantification
Source: Hum Gene Ther. 2022 Jul 13;33(13-14):765–75. doi: 10.1089/hum.2022.013 (PMC9347376; doi:10.1089/hum.2022.013)

**Figure S1. RP-HPLC separation of V937 DS virion proteins**


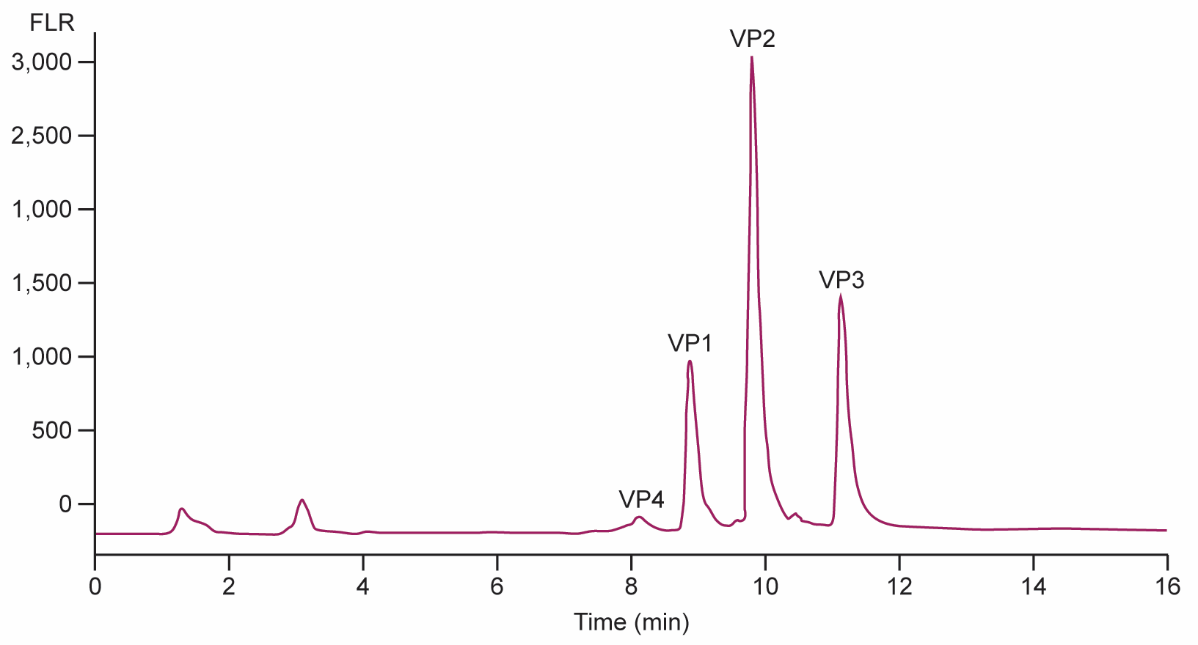

Supplement: Supplemental data [file Suppl_FigS1.docx]

**Figure S2. RP-UPLC separation of V937 DS virion proteins**


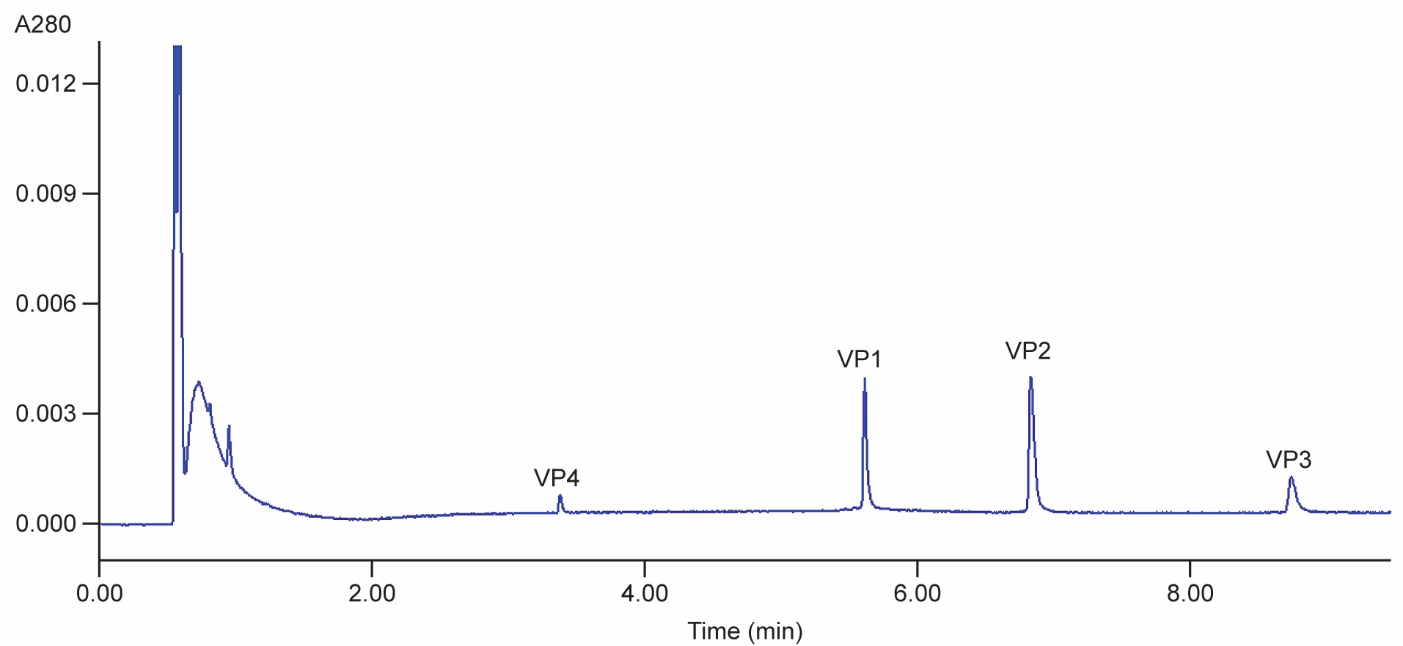

Supplement: Supplemental data [file Suppl_FigS2.docx]

**Figure S5. Overlay of chromatograms on different column temperature**


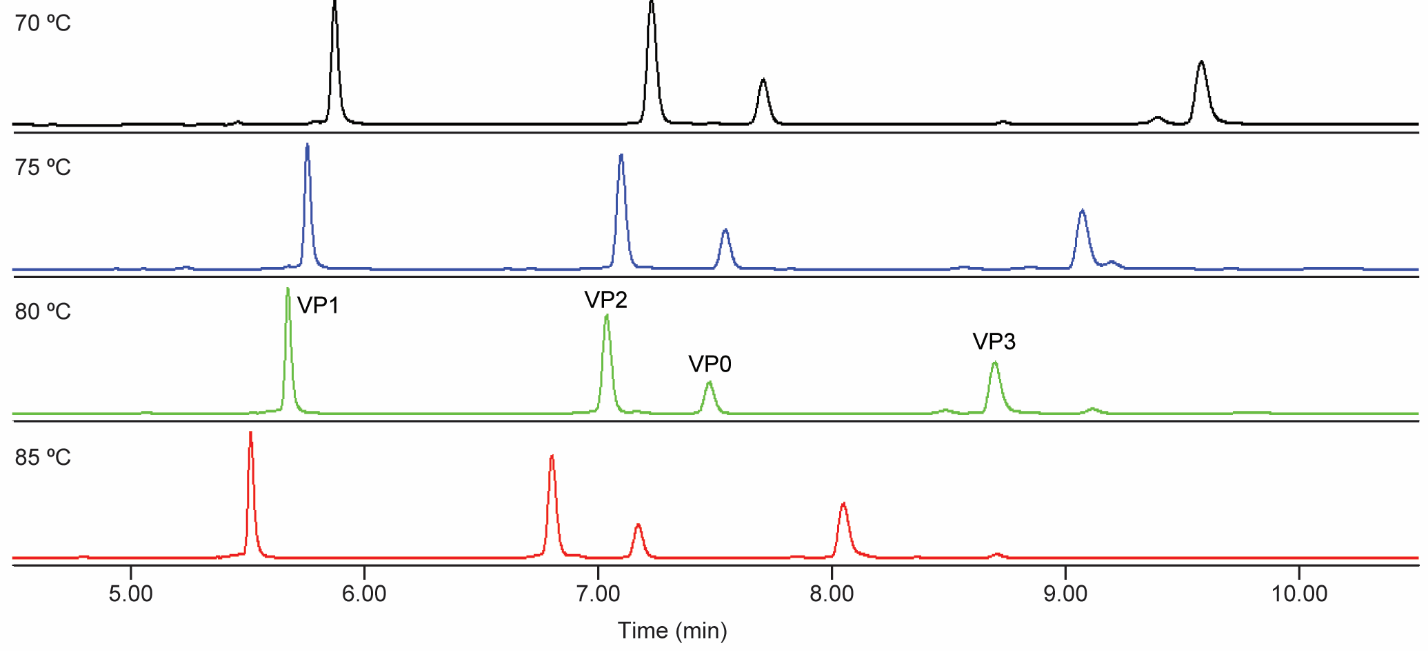

Supplement: Supplemental data [file Suppl_FigS5.docx]

**Figure S6. Overlay of chromatograms on different flow rates**


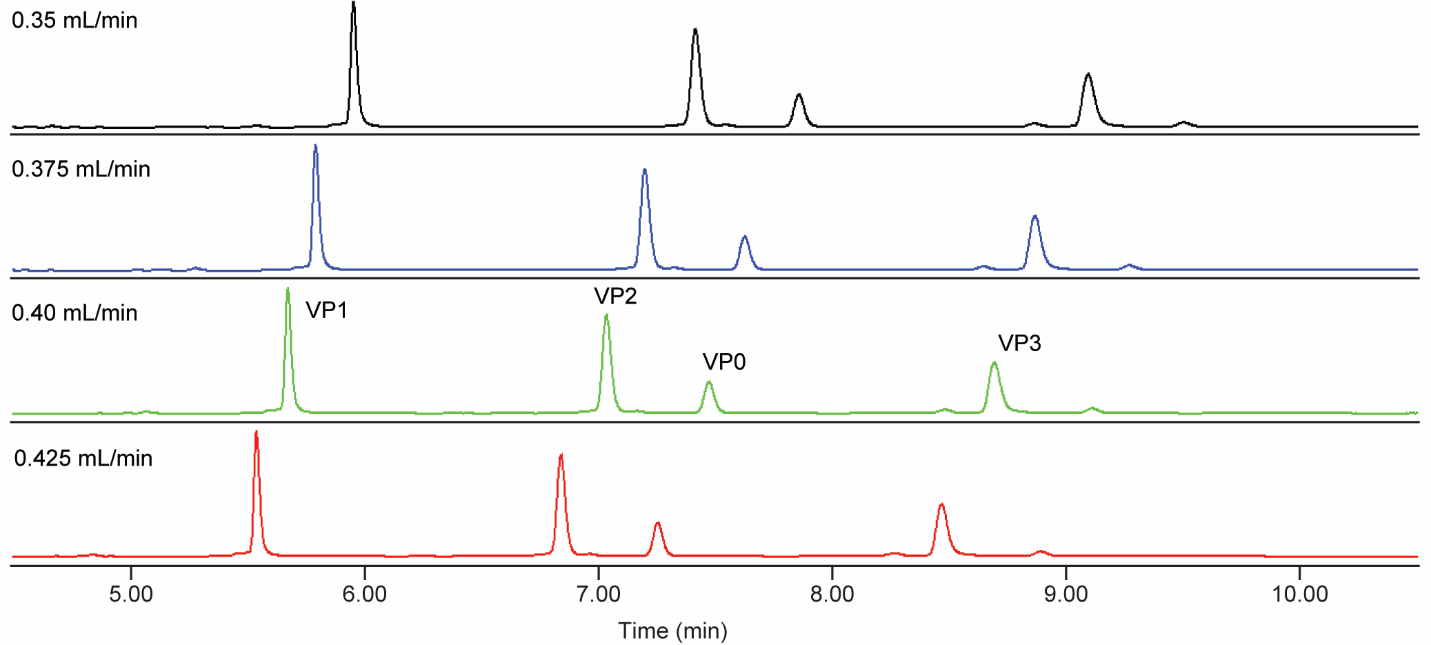

Supplement: Supplemental data [file Suppl_FigS6.docx]

**Figure S7. Separation capsid virion proteins with spiked BSA**


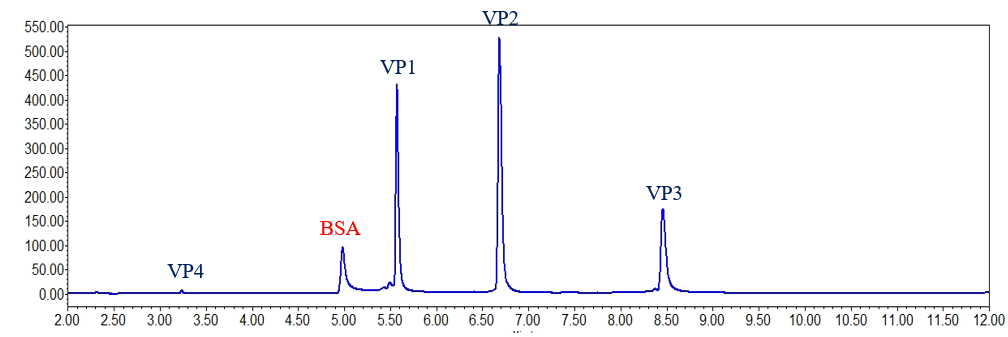

Supplement: Supplemental data [file Suppl_FigS7.docx]
