# Supplementary material for: Reverse-Phase Ultra-Performance Chromatography Method for Oncolytic Coxsackievirus Viral Protein Separation and Empty to Full Capsid Quantification
Source: Hum Gene Ther. 2022 Jul 13;33(13-14):765–75. doi: 10.1089/hum.2022.013 (PMC9347376; doi:10.1089/hum.2022.013)
Supplement: Supplemental data [file Suppl_TableS1.docx]

**Table S1a. Peak resolution of virion proteins on RP-HPLC**

| VP Peaks | Peak RT (min) | Selectivity | Resolution (R) |
| --- | --- | --- | --- |
| VP4 | 8.13 |  |  |
| VP1 | 8.74 | 1.09 | **2.04** |
| VP2 | 9.81 | 1.10 | **3.17** |
| VP3 | 11.1 | 1.14 | **4.54** |

**Table S1b. Peak resolution of virion proteins on RP-UPLC**

| VP Peaks | Peak RT (min) | Selectivity | Resolution (R) |
| --- | --- | --- | --- |
| VP4 | 4.39 |  |  |
| VP1 | 6.22 | 1.44 | **32.6** |
| VP2 | 8.51 | 1.38 | **29.9** |
| VP3 | 10.5 | 1.24 | **20.6** |

Peak resolution Rs is calculated using Equation-S1 by instrument software.

Equation-S1.

$$Rs=\frac{1.18(RT2-RT1)}{(W2+W1)}$$

RT2 and RT1: Retention time of peaks

W2 and W1: Peak width at 50% peak height
