# Supplementary material for: Reverse-Phase Ultra-Performance Chromatography Method for Oncolytic Coxsackievirus Viral Protein Separation and Empty to Full Capsid Quantification
Source: Hum Gene Ther. 2022 Jul 13;33(13-14):765–75. doi: 10.1089/hum.2022.013 (PMC9347376; doi:10.1089/hum.2022.013)

**Figure S3. Comparison of DTT treated V937 DS with untreated DS on RP-UPLC**

DTT treated V93 DS sample was obtained by adding 25 µL of 0.25M DTT solution into 100 µL V937 drug substance and incubated at 60 ^o^C for 45 minutes before RP-UPLC analysis. Native capsids chromatogram was obtained by direct injection of V937 DS on UPLC.


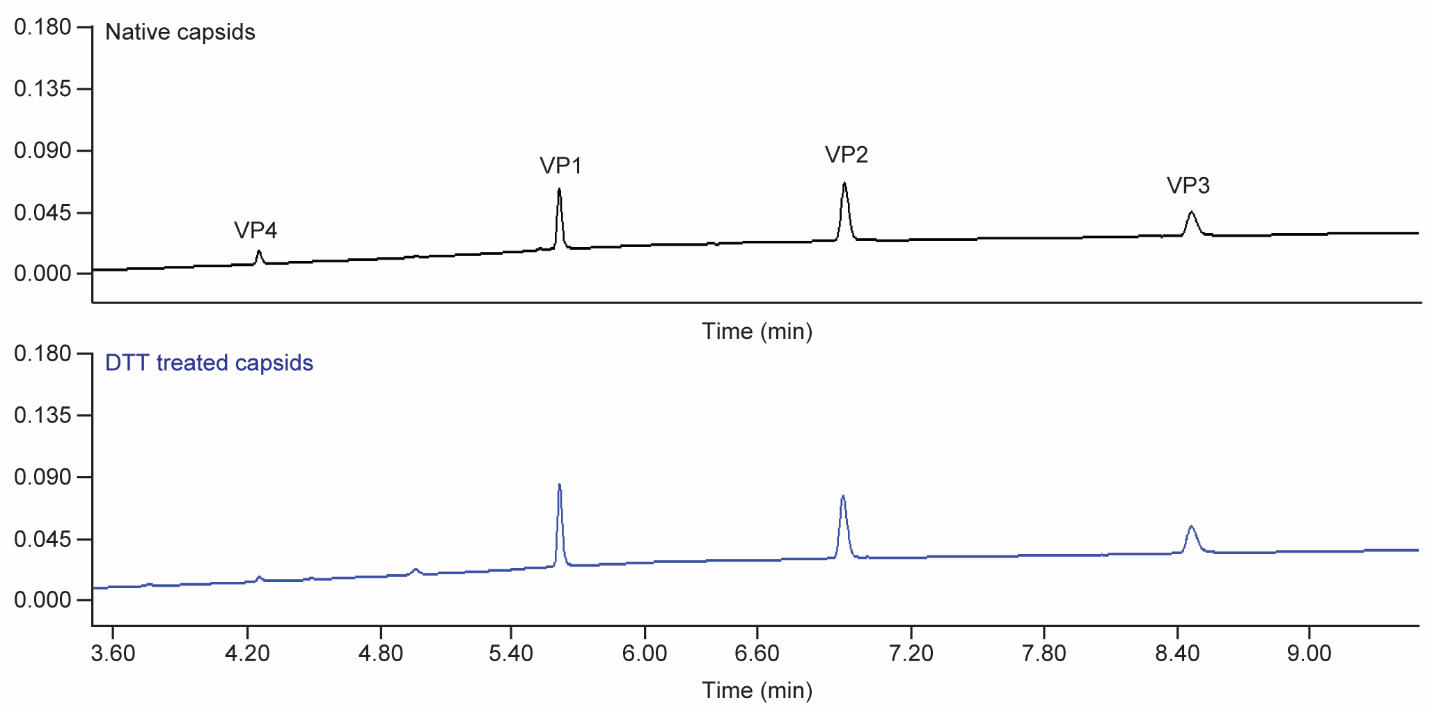

Supplement: Supplemental data [file Suppl_FigS3.docx]
