# Supplementary material for: Reverse-Phase Ultra-Performance Chromatography Method for Oncolytic Coxsackievirus Viral Protein Separation and Empty to Full Capsid Quantification
Source: Hum Gene Ther. 2022 Jul 13;33(13-14):765–75. doi: 10.1089/hum.2022.013 (PMC9347376; doi:10.1089/hum.2022.013)
Supplement: Supplemental data [file Suppl_TableS2.docx]

**Table S2. Calculated physical properties for VP1, VP2, VP3 and VP4**

| **Virion Proteins** | **VP4** | **VP1** | **VP2** | **VP3** |
| --- | --- | --- | --- | --- |
| Mw (kDa) | 7.3 | 33.2 | 29.9 | 26.5 |
| A280 Ex. Coff (M^-1^ cm^-1^) | 4470 | 44600 | 54235 | 25815 |
| Aliphatic index | 58.97 | 73.26 | 78.16 | 78.83 |
| Grand average of hydropathicity (GRAVY) | -0.815 | -0.289 | -0.143 | -0.02 |
| Number of Trp (W) |  | 4 | 6 | 3 |
| Number of Tyr (Y) | 3 | 15 | 14 | 6 |
| Number of Phe (F) | 1 | 14 | 10 | 13 |
