# Supplementary material for: Reverse-Phase Ultra-Performance Chromatography Method for Oncolytic Coxsackievirus Viral Protein Separation and Empty to Full Capsid Quantification
Source: Hum Gene Ther. 2022 Jul 13;33(13-14):765–75. doi: 10.1089/hum.2022.013 (PMC9347376; doi:10.1089/hum.2022.013)

**Figure S8. Chromatogram overlay of a Coxsackievirus full capsid (blue trace) and a sample with high empty capsid content (red trace). VP0 is not visible in the full capsid (blue trace), while only very low level of VP2 is observed in the red trace.**


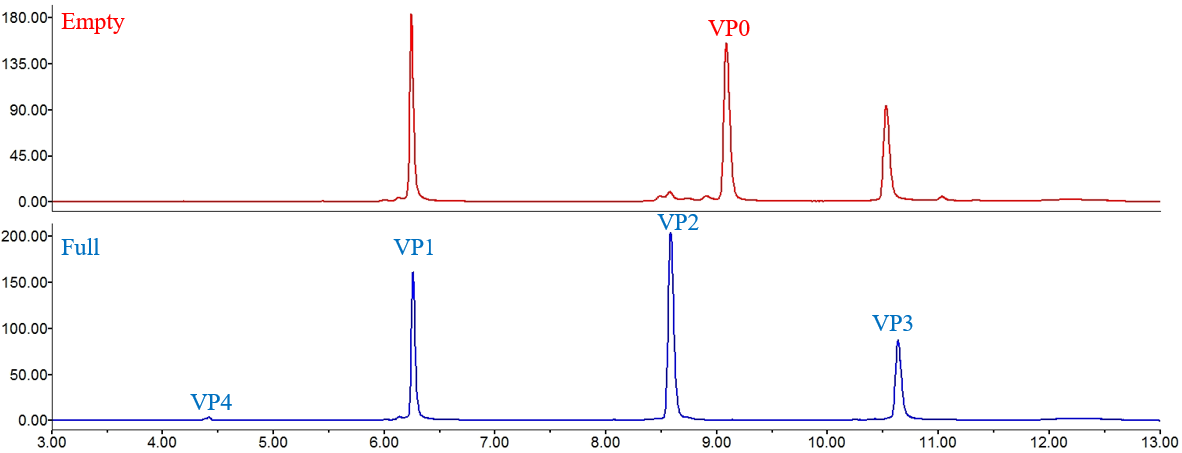

Supplement: Supplemental data [file Suppl_FigS8.docx]
