# Supplementary material for: Reverse-Phase Ultra-Performance Chromatography Method for Oncolytic Coxsackievirus Viral Protein Separation and Empty to Full Capsid Quantification
Source: Hum Gene Ther. 2022 Jul 13;33(13-14):765–75. doi: 10.1089/hum.2022.013 (PMC9347376; doi:10.1089/hum.2022.013)
Supplement: Supplemental data [file Suppl_FigS4.docx]

**Figure S4. Mass spectra for each virion protein**

**Figure S4-1. VP4 mass spectra**



**Figure S4-2.** **VP0 mass spectra**





**Figure S4-3. VP1 mass spectra**





**Figure S4-4. VP2 mass spectra**





**Figure S4-5. VP3 mass spectra**
