# Supplementary material for: Reverse-Phase Ultra-Performance Chromatography Method for Oncolytic Coxsackievirus Viral Protein Separation and Empty to Full Capsid Quantification
Source: Hum Gene Ther. 2022 Jul 13;33(13-14):765–75. doi: 10.1089/hum.2022.013 (PMC9347376; doi:10.1089/hum.2022.013)
Supplement: Supplemental data [file Suppl_TableS3.docx]

**Table S3. Peak resolution for VPs at different column temperatures**

| Column Temp (^o^C) | Peak resolution (Rs) | | |
| --- | --- | --- | --- |
|  | VP1/VP2 | VP2/VP0 | VP0/VP3 |
| 70 | 21.4 | 6.1 | 20.4 |
| 75 | 22.2 | 5.9 | 17.4 |
| **80** | **23.1** | **5.8** | **14.0** |
| 85 | 23.1 | 5.3 | 10.9 |
