# Supplementary material for: Reverse-Phase Ultra-Performance Chromatography Method for Oncolytic Coxsackievirus Viral Protein Separation and Empty to Full Capsid Quantification
Source: Hum Gene Ther. 2022 Jul 13;33(13-14):765–75. doi: 10.1089/hum.2022.013 (PMC9347376; doi:10.1089/hum.2022.013)
Supplement: Supplemental data [file Suppl_TableS4.docx]

**Table S4. Peak resolution for VPs at different flow rates**

| Flow rate (mL/min) | Peak resolution (Rs) | | |
| --- | --- | --- | --- |
|  | VP1/VP2 | VP2/VP0 | VP0/VP3 |
| 0.35 | 23.4 | 5.6 | 13.6 |
| 0.375 | 23.3 | 5.6 | 14.1 |
| **0.40** | **23.1** | **5.8** | **14.0** |
| 0.425 | 22.9 | 5.8 | 14.5 |
