# Supplementary material for: Reverse-Phase Ultra-Performance Chromatography Method for Oncolytic Coxsackievirus Viral Protein Separation and Empty to Full Capsid Quantification
Source: Hum Gene Ther. 2022 Jul 13;33(13-14):765–75. doi: 10.1089/hum.2022.013 (PMC9347376; doi:10.1089/hum.2022.013)
Supplement: Supplemental data [file Suppl_TableS5.docx]

**Table S5. Lineraity of VPs from reference standard**

| **Standard** | **Particle number/inj** | **UPLC FLR peak area** | | | | | |
| --- | --- | --- | --- | --- | --- | --- | --- |
|  |  | Total VP | Avg VP0 | Avg VP1 | Avg VP2 | Avg VP3 | Avg VP4 |
| STD-1 | 2.78E+09 | 2711929 | 5359 | 727030 | 1306452 | 662178 | 10911 |
| STD-2 | 6.95E+09 | 6493272 | 9670 | 1711557 | 3204287 | 1537811 | 29949 |
| STD-3 | 1.39E+10 | 13443037 | 18409 | 3625379 | 6599919 | 3139552 | 59779 |
| STD-4 | 3.48E+10 | 34974309 | 45264 | 9538507 | 17213260 | 8018916 | 158362 |
| STD-5 | 6.95E+10 | 70861299 | 86312 | 19836176 | 34647598 | 15958548 | 332666 |
| STD-6 | 1.04E+11 | 105435914 | 128752 | 30087650 | 50511074 | 24227102 | 481337 |
| **R^2^** | | **0.9999** | **0.9999** | **0.9998** | **0.9996** | **0.9999** | **0.9993** |
| Intercept | | -397809.6802 | 1790.022769 | -323872.555 | -13625.7 | -59384.21 | -2716.111 |
| Slope | | 0.001017993 | 1.22E-06 | 0.000290654 | 0.00048923 | 0.0002322 | 4.69E-06 |
