# Supplementary material for: Reverse-Phase Ultra-Performance Chromatography Method for Oncolytic Coxsackievirus Viral Protein Separation and Empty to Full Capsid Quantification
Source: Hum Gene Ther. 2022 Jul 13;33(13-14):765–75. doi: 10.1089/hum.2022.013 (PMC9347376; doi:10.1089/hum.2022.013)
Supplement: Supplemental data [file Suppl_TableS6.docx]

**Table S6. Sample dilutional linearity**

| INJ Vol (mL) | 0.030 | | FLR peak areas | | | | | |  |  |
| --- | --- | --- | --- | --- | --- | --- | --- | --- | --- | --- |
| Sample-A | Dilution factor | Inj Vol (mL)/Diltion factor | Total VPs | VP4 | VP1 | VP2 | VP0 | VP3 | Particle number per INJ | Sample capsid concentration (capsids/mL) |
| Dilution-15x | 15 | 0.0020 | 6839906 | 22600 | 1929127 | 3379171 | 25223 | 1483785 | 7.11E+09 | 3.55E+12 |
| Dilution-6x | 6 | 0.0050 | 16613250 | 56723 | 4675353 | 8275386 | 52559 | 3553229 | 1.67E+10 | 3.34E+12 |
| Dilution-3x | 3 | 0.0100 | 32619303 | 118373 | 9057040 | 16442743 | 101318 | 6899829 | 3.24E+10 | 3.24E+12 |
| Dilution-1.5x | 1.5 | 0.0200 | 64550956 | 237974 | 17942838 | 32248197 | 238864 | 13883083 | 6.38E+10 | 3.19E+12 |
| Neat | 1 | 0.0300 | 98045076 | 336155 | 27345723 | 48448609 | 334844 | 21579745 | 9.67E+10 | 3.22E+12 |
| STD Curve Intercept | -397809.68 | RSQ | 0.9999 | 0.9980 | 0.9998 | 1.0000 | 0.9952 | 0.9992 | 0.9999 |  |
| STD Curve Slope | 0.001017993 |  |  |  |  |  |  |  | Average | 3.3E+12 |
|  |  |  |  |  |  |  |  |  | %RSD | 4.5 |
