# Supplementary material for: Reverse-Phase Ultra-Performance Chromatography Method for Oncolytic Coxsackievirus Viral Protein Separation and Empty to Full Capsid Quantification
Source: Hum Gene Ther. 2022 Jul 13;33(13-14):765–75. doi: 10.1089/hum.2022.013 (PMC9347376; doi:10.1089/hum.2022.013)
Supplement: Supplemental data [file Suppl_TableS7.docx]

**Table S7. Retention time precision**

| Sample | INJ | Retention Time (min) | | | | |
| --- | --- | --- | --- | --- | --- | --- |
|  |  | VP4 | VP1 | VP2 | VP0 | VP3 |
| Sample-2 | INJ-1 | 4.366 | 5.689 | 7.150 | 7.625 | 8.913 |
|  | INJ-2 | 4.369 | 5.692 | 7.163 | 7.638 | 8.917 |
|  | INJ-3 | 4.373 | 5.694 | 7.163 | 7.637 | 8.913 |
|  | Avg | 4.369 | 5.692 | 7.159 | 7.633 | 8.914 |
|  | %RSD | 0.08 | 0.04 | 0.1 | 0.09 | 0.03 |
| Sample-3 | INJ-1 | 4.370 | 5.693 | 7.164 | 7.647 | 8.929 |
|  | INJ-2 | 4.375 | 5.699 | 7.170 | 7.656 | 8.933 |
|  | INJ-3 | 4.373 | 5.698 | 7.163 | 7.644 | 8.921 |
|  | Avg | 4.373 | 5.697 | 7.166 | 7.649 | 8.928 |
|  | %RSD | 0.06 | 0.06 | 0.05 | 0.08 | 0.07 |
| Avg | | 4.373 | 5.696 | 7.166 | 7.642 | 8.925 |
| %RSD | | 0.1 | 0.08 | 0.13 | 0.18 | 0.17 |
