# Supplementary material for: Reverse-Phase Ultra-Performance Chromatography Method for Oncolytic Coxsackievirus Viral Protein Separation and Empty to Full Capsid Quantification
Source: Hum Gene Ther. 2022 Jul 13;33(13-14):765–75. doi: 10.1089/hum.2022.013 (PMC9347376; doi:10.1089/hum.2022.013)
Supplement: Supplemental data [file Suppl_TableS8.docx]

**Table S8. Peak area and measurement precision**

| Sample-2 | FLR peak area | | | | | | | Empty/Full | Particle number (/mL) |
| --- | --- | --- | --- | --- | --- | --- | --- | --- | --- |
|  | VP4 | VP1 | VP2 | VP0 | VP3 | Total | VP2+VP4 | VP0/(VP2+VP4) |  |
| INJ-1 | 158725 | 10247361 | 17517712 | 46682 | 8489843 | 36460323 | 17676437 | 0.00264 | 1.81E+12 |
| INJ-2 | 162856 | 10640280 | 17950269 | 46717 | 8794348 | 37594470 | 18113125 | 0.00258 | 1.87E+12 |
| INJ-3 | 153701 | 11786662 | 18409217 | 49979 | 9350333 | 39749892 | 18562918 | 0.00269 | 1.97E+12 |
| Avg | 158427 | 10891434 | 17959066 | 47793 | 8878175 | 37934895 | 18117493 | 0.00264 | 1.88E+12 |
| %RSD | 2.89 | 7.34 | 2.48 | 3.96 | 4.91 | 4.4 | 2.45 | 2.15 | 4.36 |

| Sample-3 | FLR peak area | | | | | | | Empty/Full | Particle number (/mL) |
| --- | --- | --- | --- | --- | --- | --- | --- | --- | --- |
|  | VP4 | VP1 | VP2 | VP0 | VP3 | Total | VP2+VP4 | VP0/(VP2+VP4) |  |
| INJ-1 | 330522 | 22202996 | 38408783 | 121991 | 18010071 | 79074363 | 38739305 | 0.00315 | 3.90E+12 |
| INJ-2 | 326236 | 22666758 | 38676791 | 121767 | 18144721 | 79936273 | 39003027 | 0.00312 | 3.95E+12 |
| INJ-3 | 334445 | 21932179 | 39088999 | 124316 | 17962501 | 79442440 | 39423444 | 0.00315 | 3.92E+12 |
| Avg | 330401 | 22267311 | 38724858 | 122691 | 18039098 | 79484359 | 39055259 | 0.00314 | 3.92E+12 |
| %RSD | 1.24 | 1.67 | 0.88 | 1.15 | 0.52 | 0.54 | 0.88 | 0.54 | 0.54 |
