# Supplementary material for: Reverse-Phase Ultra-Performance Chromatography Method for Oncolytic Coxsackievirus Viral Protein Separation and Empty to Full Capsid Quantification
Source: Hum Gene Ther. 2022 Jul 13;33(13-14):765–75. doi: 10.1089/hum.2022.013 (PMC9347376; doi:10.1089/hum.2022.013)
Supplement: Supplemental data [file Suppl_TableS9.docx]

**Table S9. List of standard and spike sample**

| Sample | Total capsid (capsids/mL) | Empty/full ratio | %Empty | %Full | Full capsid (capsids/mL) | Empty particle (capsids/mL) |
| --- | --- | --- | --- | --- | --- | --- |
| Standard | 1.39E+12 | 0.00252 | 0.25 | 99.75 | 1.39E+12 | 3.50E+09 |
| Sample-B | 3.66E+12 | 0.523 | 34.3 | 65.67 | 2.40E+12 | 1.26E+12 |
