# Supplementary material for: Reverse-Phase Ultra-Performance Chromatography Method for Oncolytic Coxsackievirus Viral Protein Separation and Empty to Full Capsid Quantification
Source: Hum Gene Ther. 2022 Jul 13;33(13-14):765–75. doi: 10.1089/hum.2022.013 (PMC9347376; doi:10.1089/hum.2022.013)
Supplement: Supplemental data [file Suppl_TableS10.docx]

**Table S10. Spiked sample preparatiuon**

| Mix sample | Standard Vol (uL) | Sample-B Vol (uL) | Total Vol (uL) | Standard/ Sample-1 Ratio (v/v) | Standard dilution | Sample-1 dilution |
| --- | --- | --- | --- | --- | --- | --- |
| Mix-1 | 40 | 40 | 80 | 1 | 2 | 2 |
| Mix-2 | 60 | 6 | 66 | 10 | 1.1 | 11 |
| Mix-3 | 75 | 3 | 78 | 25 | 1.04 | 26 |
| Mix-4 | 80 | 2 | 82 | 40 | 1.025 | 41 |
