# Supplementary material for: Reverse-Phase Ultra-Performance Chromatography Method for Oncolytic Coxsackievirus Viral Protein Separation and Empty to Full Capsid Quantification
Source: Hum Gene Ther. 2022 Jul 13;33(13-14):765–75. doi: 10.1089/hum.2022.013 (PMC9347376; doi:10.1089/hum.2022.013)
Supplement: Supplemental data [file Suppl_TableS11.docx]

**Table S11. Theoretical values for each sample**

| Sample | Full capsid (capsids/mL) from Standard | Empty capsid (capsids/mL) from Standard | Full capsid (capsids/mL) from Sample-B | Empty capsid (capsids/mL) from Sample-B | All full capsid (capsids /mL) | All empty capsid (capsids /mL) | Total capsid (capsids /mL) | Empty/full ratio |
| --- | --- | --- | --- | --- | --- | --- | --- | --- |
| Mix-1 | 6.93E+11 | 1.75E+09 | 1.20E+12 | 6.28E+11 | 1.89E+12 | 6.29E+11 | 2.52E+12 | 0.3323 |
| Mix-2 | 1.26E+12 | 3.18E+09 | 2.18E+11 | 1.14E+11 | 1.48E+12 | 1.17E+11 | 1.60E+12 | 0.0793 |
| Mix-3 | 1.33E+12 | 3.37E+09 | 9.24E+10 | 4.83E+10 | 1.43E+12 | 5.16E+10 | 1.48E+12 | 0.0362 |
| Mix-4 | 1.35E+12 | 3.42E+09 | 5.86E+10 | 3.06E+10 | 1.41E+12 | 3.40E+10 | 1.45E+12 | 0.0241 |
