# Supplementary material for: Reverse-Phase Ultra-Performance Chromatography Method for Oncolytic Coxsackievirus Viral Protein Separation and Empty to Full Capsid Quantification
Source: Hum Gene Ther. 2022 Jul 13;33(13-14):765–75. doi: 10.1089/hum.2022.013 (PMC9347376; doi:10.1089/hum.2022.013)
Supplement: Supplemental data [file Suppl_TableS12.docx]

**Table S12. Measured capsid particle concentration and empty/full ratio for spiked samples**

| Sample | INJ Vol (uL) | FLR peak area | | | | | | Measured total capsids(capsids/ mL) | Measured empty/full |
| --- | --- | --- | --- | --- | --- | --- | --- | --- | --- |
|  |  | Total VPs | VP4 | VP1 | VP2 | VP0 | VP3 | NA | VP0/(VP2+VP4) |
| Mix-1 | 20 | 52146937 | 161691 | 15449147 | 18171396 | 6006106 | 12358597 | 2.58E+12 | 0.3276 |
| Mix-2 | 20 | 33715465 | 135066 | 9576971 | 15010190 | 1083557 | 7909681 | 1.68E+12 | 0.0715 |
| Mix-3 | 20 | 31598552 | 130258 | 8923914 | 14565979 | 531953 | 7446449 | 1.57E+12 | 0.0362 |
| Mix-4 | 20 | 30542592 | 130253 | 8606126 | 14326761 | 342040 | 7137413 | 1.52E+12 | 0.0237 |
| RSQ | 0.999934878 |  |  |  |  |  |  |  |  |
| Intercept | -397809.9411 |  |  |  |  |  |  |  |  |
| Slope | 0.001017993 |  |  |  |  |  |  |  |  |
