# Supplementary material for: Reverse-Phase Ultra-Performance Chromatography Method for Oncolytic Coxsackievirus Viral Protein Separation and Empty to Full Capsid Quantification
Source: Hum Gene Ther. 2022 Jul 13;33(13-14):765–75. doi: 10.1089/hum.2022.013 (PMC9347376; doi:10.1089/hum.2022.013)
Supplement: Supplemental data [file Suppl_TableS13.docx]

**Table S13. Comparison of empty/full ratio of batches from two different processess**

| Process | Batch | VP0/VP2 (%) |
| --- | --- | --- |
| Process-1 | Experiment-1 | 32.7 |
|  | Experiment-2 | 22.4 |
|  | Experiment-3 | 20.9 |
| Process-2 | Experiment-4 | 2.07 |
|  | Experiment-5 | 3.62 |
|  | Experiment-6 | 2.11 |
