# Supplementary material for: Reverse-Phase Ultra-Performance Chromatography Method for Oncolytic Coxsackievirus Viral Protein Separation and Empty to Full Capsid Quantification
Source: Hum Gene Ther. 2022 Jul 13;33(13-14):765–75. doi: 10.1089/hum.2022.013 (PMC9347376; doi:10.1089/hum.2022.013)
Supplement: Supplemental data [file Suppl_TableS14.docx]

**Table S14. VP0/VP2 ratios from five V937 DS batches**

| DS Batch |  | FLR peak area | |  |
| --- | --- | --- | --- | --- |
| Btach-1 | Injection | VP2 | VP0 | % (VP0/VP2) |
|  | INJ1 | 19480510 | 31355 | 0.16 |
|  | INJ2 | 19557871 | 31506 | 0.16 |
|  | Average | 19519191 | 31431 | 0.16 |
|  | INJ1/INJ2 | 1 | 1 | 1 |
| Btach-2 | Injection | VP2 | VP0 | % (VP0/VP2) |
|  | INJ1 | 20382730 | 33973 | 0.17 |
|  | INJ2 | 20170658 | 33266 | 0.16 |
|  | Average | 20276694 | 33620 | 0.17 |
|  | INJ1/INJ2 | 1.01 | 1.02 | 1.01 |
| Btach-3 | Injection | VP2 | VP0 | % (VP0/VP2) |
|  | INJ1 | 40723107 | 172393 | 0.42 |
|  | INJ2 | 41118599 | 154715 | 0.38 |
|  | Average | 40920853 | 163554 | 0.4 |
|  | INJ1/INJ2 | 0.99 | 1.11 | 1.13 |
| Btach-4 | Injection | VP2 | VP0 | % (VP0/VP2) |
|  | INJ1 | 24591498 | 173187 | 0.7 |
|  | INJ2 | 25512906 | 185280 | 0.73 |
|  | Average | 25052202 | 179234 | 0.72 |
|  | INJ1/INJ2 | 0.96 | 0.93 | 0.97 |
| Btach-5 | Injection | VP2 | VP0 | % (VP0/VP2) |
|  | INJ1 | 19193956 | 78540 | 0.41 |
|  | INJ2 | 17985849 | 78327 | 0.44 |
|  | Average | 18589903 | 78434 | 0.42 |
|  | INJ1/INJ2 | 1.07 | 1 | 0.94 |

| Scale-up Batch | Batch-1 | Batch-2 | Batch-3 | Batch-4 | Batch-5 |
| --- | --- | --- | --- | --- | --- |
| Avg VP0/VP2 | 0.16 | 0.17 | 0.40 | 0.72 | 0.42 |
